# Effect of hydrothermal treatment of titanium in high concentration of AgNO _3_ solution on surface morphology and roughness

**DOI:** 10.12688/f1000research.79542.1

**Published:** 2022-02-23

**Authors:** Sunarso Sunarso, Raihan Jazmi Hares Putra, Citra Fragrantia Theodorea, Azizah Intan Pangesty

**Affiliations:** 1 Department of Dental Materials, Faculty of Dentistry, Universitas Indonesia, Jakarta, 10430, Indonesia; 2 Undergraduate Program, Department of Metallurgy and Materials Engineering, Faculty of Engineering, Universitas Indonesia, Depok, 16425, Indonesia; 3 Department of Oral Biology, Faculty of Dentistry, Universitas Indonesia, Jakarta, 10430, Indonesia; 4 Department of Metallurgy and Materials Engineering, Faculty of Engineering, Universitas Indonesia, Depok, 16425, Indonesia

**Keywords:** Silver nitrate, titanium, hydrothermal, surface morphology, roughness

## Abstract

Development of silver (Ag) modified titanium (Ti) as an antibacterial dental implant has recently been growing. Ag demonstrated an excellent antibacterial property without the risk of bacterial resistance. Hydrothermal treatment using AgNO _3_ solution is one of the facile and promising methods to modify Ti surface with Ag. However, the effect of high AgNO _3_ concentration and the absent of a toxic reduction agent has not been clearly studied. In this study, Ti surface was hydrothermally treated in 0.01 mol/L and 0.1 mol/L AgNO _3_ solutions at 150 ^o^C for 24 hours. Analysis of surface morphology using scanning electron microscopy with energy dispersive X-ray analysis suggested the formation of non-homogenous Ag coating with a tendency to be aggregated and thicken with the increase of AgNO _3 _concentration. The Ag coating deposited on Ti surface were composed of mainly metallic and some oxide forms. Surface roughness of all AgNO _3_ treated Ti surface was comparable based on the analysis of surface roughness parameter. In conclusion, hydrothermal treatment of Ti surface in solely AgNO _3 _solution at high concentration produced non-homogenous Ag coating on its surface without significantly changed surface roughness.

Keywords: Silver nitrate, titanium, hydrothermal, surface morphology, roughness

## Introduction

Titanium (Ti) has widely been used clinically for dental implants due to their excellent mechanical properties, biocompatibility and osteoconductivity. ^1^^,^^2^ Development of Ti implant for dental application is still challenging. Dental implant not only requires osseointegration capability but also antibacterial property. ^3^ Titanium demonstrated satisfactory osseointegration clinically. However, its antibacterial capability is lacking.

Silver coating has emerged as an alternative to prepare antibacterial titanium surface. ^4^ Silver is considered a promising element to prevent and combat implant-related infection. Its main advantage is that it would not induce bacterial resistance. ^5^ Silver is coated onto Ti surface often in the form of particles by immersion in AgNO _3_ solution mixed with a reduction agent. ^6^^,^^7^ This results in silver particulates being deposited on the Ti surface often in nanoscale, thus called silver nanoparticles (AgNPs). The use of reduction agents is problematic because they are often toxic chemicals such as Sodium borohydride _,_ ammonium formate and hydrazine. ^8^^,^^9^ A recent study has been conducted to coat Ti surface with silver under hydrothermal without the need for reduction agents. ^10^

Direct Ag coating onto Ti surface using hydrothermal has not been clearly described especially in high concentration of AgNO _3_ solutions and without the addition of toxic reduction chemicals. Therefore, this study aimed to coat the Ti surface with Ag particles using hydrothermal using solely AgNO _3_ solution. Surface morphology including the distribution of the Ag coating and the change in surface roughness were then evaluated.

## Methods

### Sample preparation

Two Ti plates (Maximus Guard, Tokopedia) with a size of 10 cm × 10 cm and thickness of 1 mm were cut into 10 mm × 10 mm using a diamond cutter. A total of fifteen Ti plates (10 mm × 10 mm) were used in this study. The samples were washed ultrasonically with acetone, ethanol, and distilled water before drying. Silver nitrate (AgNO _3_, Merck) solutions with a concentration of 0.01 mol/L and 0.1 mol/L were prepared. Titanium samples were immersed in a 100 ml-size Teflon container with 25 ml of AgNO _3_ solutions, which was then placed into a hydrothermal vessel (FBA_Lab, Tokopedia). The hydrothermal vessel was heated in an oven at 150 ^o^C for 24 hours. After hydrothermal treatment, the samples were washed with ethanol three times before drying.

### Surface chemical composition, morphology and roughness

The elemental composition of titanium surface samples was examined using energy dispersive spectroscopy (Oxford instruments, UK) and analyzed using Oxford Aztect software. Scanning electron microscope (SEM) (Thermoscientific Quanta 650) (accelerating voltage (HV): 12kV, Secondary electron (SE), working distance (WD): 10.3-10.4mm) was used to evaluate the morphology of surface before and after hydrothermal. Surface roughness of titanium samples before and after hydrothermal treatment are measured using roughness tester (Surtronic S128) (Sampling length (l): 7 mm, cut-off (λc)/Type: 0.25 mm/2CR, Range: 100 μm). The average values of surface roughness were calculated using Microsoft excel spreadsheet software.

## Results and discussion

**Figure 1.**
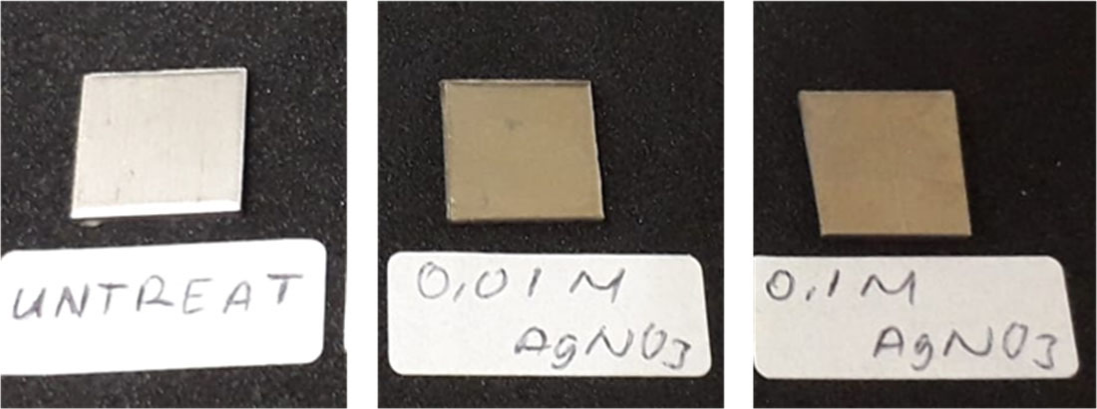
Photo of untreated Ti, Ag coated Ti from 0.01 mol/L AgNO _3_, and Ag coated Ti from 0.1 mol/L AgNO _3_.

**Figure 2.**
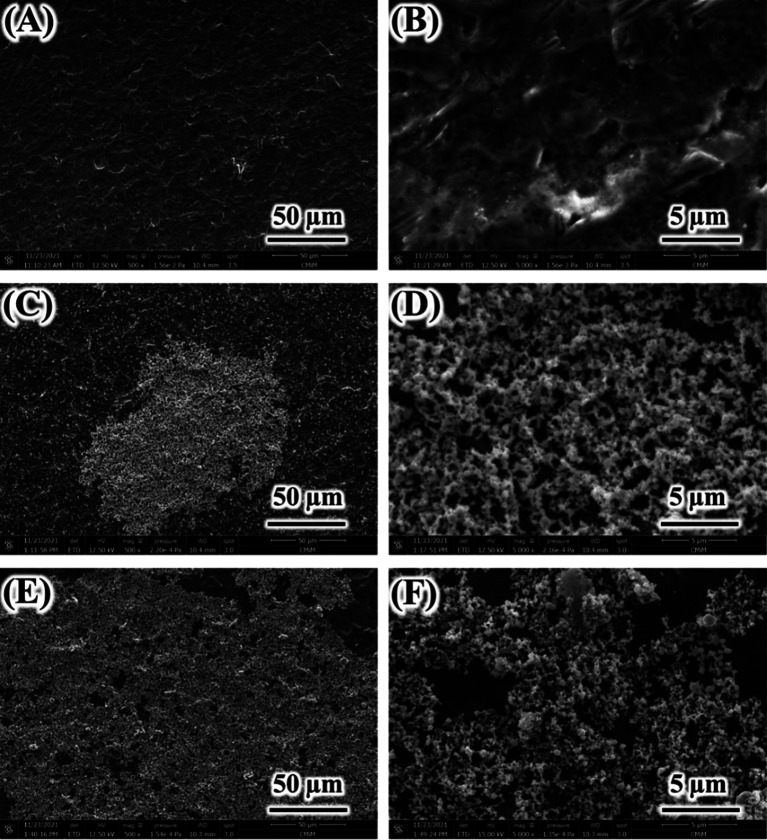
Scanning electron microscope images of untreated Ti (A and B), Ag coated Ti from 0.01 mol/L AgNO _3_ (C and D), and Ag coated Ti from 0.1 mol/L AgNO _3_ (E and F). ^14^

**Figure 3.**
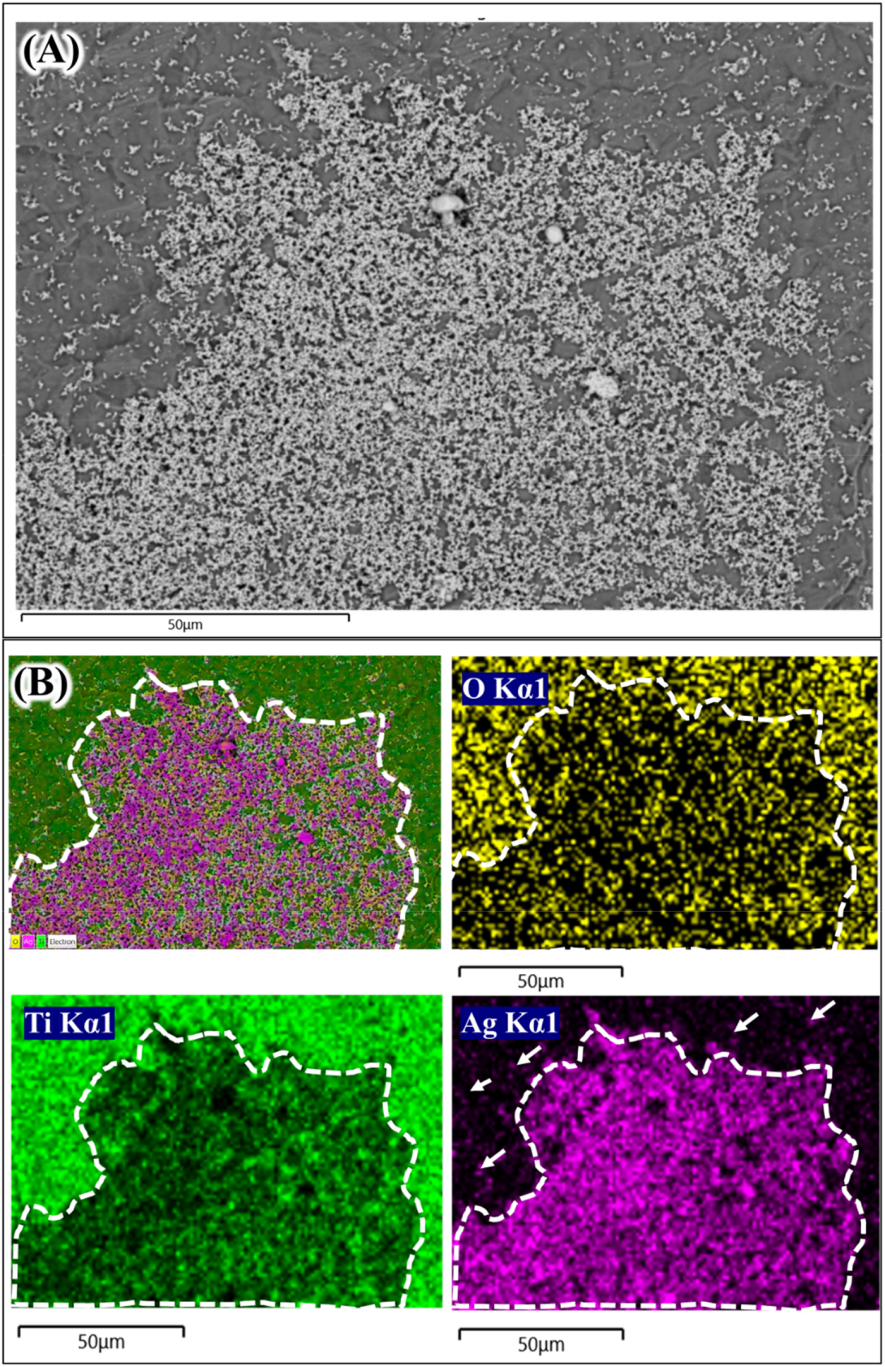
Scanning electron microscope image (A) and energy dispersive X-ray analysis element mapping images of Ag coated Ti from 0.01 mol/L AgNO _3_ (B). ^14^

**Figure 4.**
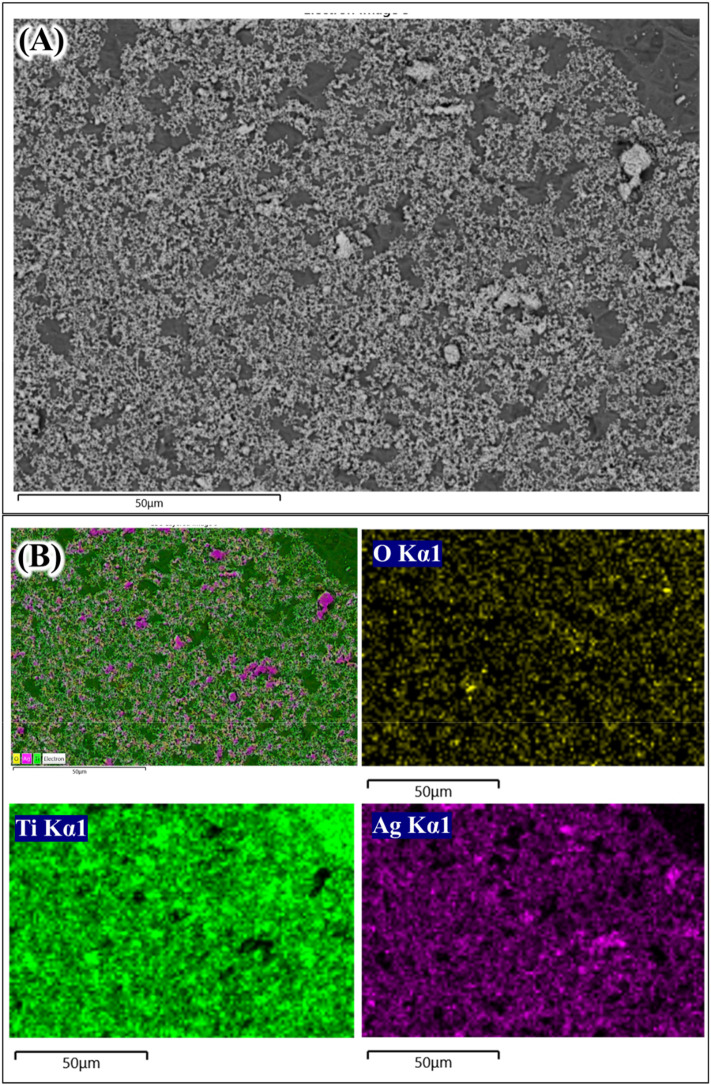
Scanning electrom microscope image (A) and energy dispersive X-ray analysis element mapping images of Ag coated Ti from 0.1 mol/L AgNO _3_ (B). ^14^

**Figure 5.**
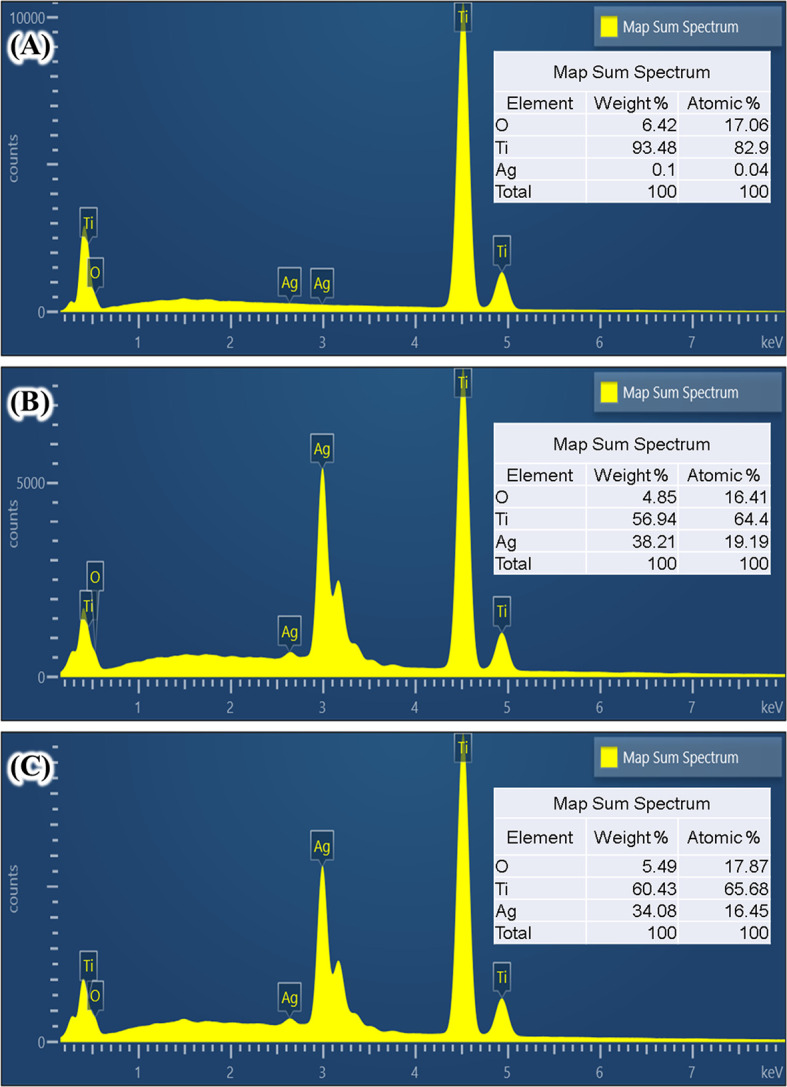
Element composition of untreated Ti (A), Ag coated Ti from 0.01 mol/L AgNO _3_ (B), and Ag coated Ti from 0.1 mol/L AgNO _3_ (C) obtained from energy dispersive X-ray analysis. ^14^

Figures 1 and 2 show the photographs and SEM images of Ti surface before and after hydrothermal in AgNO _3_ solutions. Bright particles were observed on Ti surface hydrothermally treated in 0.01 mol/L and 0.1 mol/L AgNO _3_. An area showed more concentrated particles, which may indicate the agglomeration. At higher concentration of AgNO _3_ (0.1 mol/L), that concentrated area was larger ( Figure 2). The elemental analysis from the surface using energy dispersive X-ray analysis (EDX) indicated that those particles are Ag ( Figures 3- 5). Many methods have been developed to coat Ag into Ti surface both in the form of particles or ions. ^4^^,^^11^

The formation of silver particles on Ti surface under hydrothermal from AgNO _3_ solution was still unclear in this study. Several different mechanisms may be responsible for how Ag could be deposited on Ti surface from AgNO _3_ under hydrothermal treatment. One possible way is through thermal decomposition. ^12^ The deposition of Ag particles from only AgNO _3_ solution up to 75 μmol/L under hydrothermal conditions was recently reported. ^10^ However, the exact mechanism on how Ag particles could be deposited on Ti surface was not clearly described. AgNO _3_ was also reported to transform into Ag nanoparticles under hydrothermal condition at 121 ^o^C. ^13^ Hydroxyl ions exist on Ti oxide layer may also play a role on the Ag particles growth on its surface. ^6^ It is known that Ti surface naturally forms thin oxide layer which contain Ti-OH groups on its outmost part. Figure 2 has confirmed the formation of Ag particles on Ti surface both in 0.01 mol/L and 0.1 mol/L AgNO _3_ solutions. The Ag particles were also observed in the solution after hydrothermal (solution turned darkish color). The Ag particles seem to be non-homogenously distributed on Ti surface. As explained above, there is an area which contain thicker aggregated Ag particles masking the Ti surface. The concentrated Ag area was found to be larger in 0.1 mol/L AgNO _3_ than that in 0.01 mol/L AgNO _3_. These findings suggest that at high concentration of AgNO _3_, the Ag coating tends to be aggregated and thicker, thus the use of a lower concentration might be preferred.

The next question is that whether the Ag coating deposited on Ti surface is in the metallic or oxide forms. One way to find this is using the EDX elemental mapping to the aggregated Ag coating. Figures 3 and 4 show the elemental mapping of Ag coated Ti prepared from 0.01 mol/L and 0.1 mol/L AgNO _3_ solutions. ^14^ In Figure 3, a thick Ag coating area (white dash line) shows a very strong purple color compared to the area in which less Ag coating was observed (white arrows). In Ti and oxygen (O) element mapping (green color and yellow color respectively), the Ag coating area was darker compared to the rest. The O elemental mapping provided very important data about the deposited Ag coating. The darker area (white dash line; Figure 3) in O elemental mapping indicated that that area was composed mostly of metallic Ag. Ag oxide also existed since the bright yellow color was also observed sporadically inside the white dash line ( Figure 3; O Kα1). A similar trend was shown in Figure 4 where the Ag aggregate coating is larger. The thick Ag coating was most likely composed mainly from metallic Ag and smaller portion of Ag _2_O

**Figure 6.**
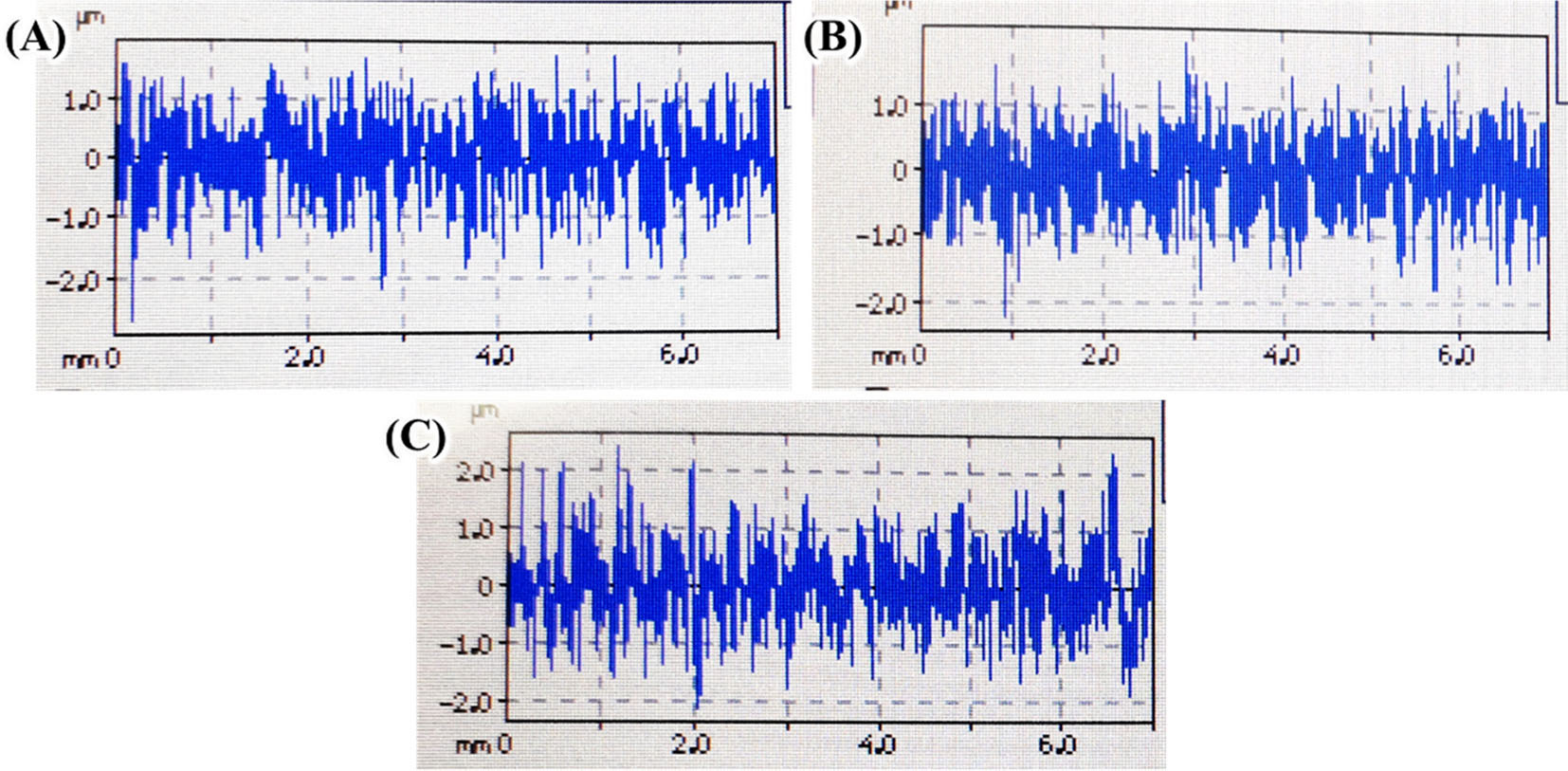
Representative of surface roughness texture of untreated Ti (A), Ag coated Ti from 0.01 mol/L AgNO _3_ (B), and Ag coated Ti from 0.1 mol/L AgNO _3_ (C) generated from roughness tester. ^16^

Surface treatment often changes surface roughness. ^15^ The change in surface roughness might alter the biological performance of Ti implant. Therefore, it is necessary to evaluate whether the current method of Ag coating changed the surface roughness of Ti surface. Surface roughness parameters roughness average (Ra), maximum profile peak height (Rp), maximum profile valley depth (Rv), and mean roughness depth (Rz) were measured from all sample surfaces. Surface roughness texture of the sample surfaces were shown ^15^ in Figure 6. ^16^ The surface texture of all Ti samples before and after Ag coating were comparable. This data suggests that no significant changes were observed on Ti surface after Ag coating ( Table 1). Comparison of SEM images between Ag coating and untreated Ti surfaces ( Figure 2) also support surface texture data. The Ti substrate in which Ag coating deposited was found to be comparable ( Figure 2A and B).

**Table 1.** Surface roughness parameter obtained from roughness tester. The values were calculated from three independent samples. ^16^

Samples	Surface roughness parameter							
	Roughness average (μm)		Maximum profile peak height (μm)		Maximum profile valley depth (μm)		Roughness depth (μm)	
	Mean	SD	Mean	SD	Mean	SD	Mean	SD
Untreated Ti	0.44	0.01	1.38	0.09	1.45	0.05	2.84	0.04
Ag-Ti 0.01	0.44	0.03	1.34	0.12	1.39	0.08	2.73	0.20
Ag-Ti 0.1	0.48	0.02	1.48	0.04	1.45	0.05	2.94	0.02

Table 1 shows the quantitative values of surface parameter obtained from the roughness tester. The surface Ra and Rv values of all samples were relatively similar which indicating its comparable average surface height and deepest valley of the substrate. A slight increase of Rp and Rz values were recorded in Table 1. Surface roughness Rp shows the maximum peak height which come from the Ag coating aggregate and was larger when a solution of 0.1 mol/L AgNO _3_ was used. The slight increase of Rp was followed by slight increase of Rz. Taken together, all surface roughness parameter demonstrated comparable values between untreated Ti (UnTi) and Ag coated Ti samples. This result suggests that hydrothermal treatment of Ti in 0.1 mol/L (Ag-Ti 0.1) and 0.01 mol/L AgNO _3_ (Ag-Ti 0.01) did not cause a notable change in the surface roughness.

## Conclusion

This study reported the effect of hydrothermal treatment of Ti surface in AgNO _3_ solution on the surface morphology and roughness. After hydrothermal treatment, an Ag coating was observed in all treated Ti surfaces. EDX mapping suggests that Ag coating composed of mainly metallic Ag and in smaller quantities, Ag oxide. Using a higher AgNO _3_ solution concentration resulted in more Ag aggregates that mask Ti surface, creating a non-homogenous coating. Surface roughness of treated Ti surface did not change significantly when coated. Nevertheless, a slight increase of Rp and Rz was observed; this might be due to Ag coating aggregates.

## Data availability

### Underlying data

Figshare: SEM and EDX https://doi.org/10.6084/m9.figshare.17159234 ^14^

This project contains the following underlying data:

- RAW SEM image (Fig 2A).jpg
- RAW SEM image (Fig 2B).jpg
- RAW SEM image (Fig 2C).jpg
- RAW SEM image (Fig 2D).jpg
- RAW SEM image (Fig 2E).jpg
- RAW SEM image (Fig 2F).jpg
- RAW EDX Mapping Data for 0.01M AgNO _3_ treated Ti (Fig. 3)
- RAW EDX Mapping Data for 0.1M AgNO _3_ treated Ti (Fig. 4)
- RAW EDX Map sum element spectrum for untreated Ti (Fig. 5A)
- RAW EDX Map sum element spectrum for 0.01M treated Ti (Fig. 5B)
- RAW EDX Map sum element spectrum for 0.1M treated Ti (Fig. 5C)

Figshare: Surface roughness https://doi.org/10.6084/m9.figshare.17215958 ^16^

This project contains the following underlying data:

- Output files for roughness testing
  o 0.01 M-1_1.jpg, 0.01 M-1_2.jpg, 0.01 M-1_3.jpg, 0.01 M-1_4.jpg (Roughness testing for 0.01 M AgNO _3_ treated Ti (specimen 1))
  o 0.01 M-2_1.jpg, 0.01 M-2_2.jpg, 0.01 M-2_3.jpg, 0.01 M-2_4.jpg (Roughness testing for 0.01 M AgNO _3_ treated Ti (specimen 2))
  o 0.01 M-3_1.jpg, 0.01 M-3_2.jpg, 0.01 M-3_3.jpg, 0.01 M-3_4.jpg (Roughness testing for 0.01 M AgNO _3_ treated Ti (specimen 3))
  o 0.1 M-1_1.jpg, 0.1 M-1_2.jpg, 0.1 M-1_3.jpg, 0.1 M-1_4.jpg (Roughness testing for 0.1 M AgNO _3_ treated Ti (specimen 1))
  o 0.1 M-2_1.jpg, 0.1 M-2_2.jpg, 0.1 M-2_3.jpg, 0.1 M-2_4.jpg (Roughness testing for 0.1 M AgNO _3_ treated Ti (specimen 2))
  o 0.1 M-3_1.jpg, 0.1 M-3_2.jpg, 0.0 M-3_3.jpg, 0.1 M-3_4.jpg (Roughness testing for 0.1 M AgNO _3_ treated Ti (specimen 3))
  o UnTi 1_1.jpg, UnTi 1_2.jpg, UnTi 1_3.jpg, UnTi 1_4.jpg (Roughness testing for untreated Ti (specimen 1))
  o UnTi 2_1.jpg, UnTi 2_2.jpg, UnTi 2-2_3.jpg, UnTi 2_4.jpg (Roughness testing for untreated Ti (specimen 2))
  o UnTi 3_1.jpg, UnTi 3_2.jpg, 0.0 UnTi 3_3.jpg, UnTi 3_4.jpg (Roughness testing for untreated Ti (specimen 3))

### Extended data

Figshare: SEM and EDX https://doi.org/10.6084/m9.figshare.17159234 ^14^

This project contains the following extended data:

- Photograph Fig 1.jpg

RAW EDX Mapping for untreated Ti

Figshare: Surface roughness https://doi.org/10.6084/m9.figshare.17215958 ^16^

This project contains the following extended data:

- Summary roughness.xlsx (Aggregated data from surface roughness testing)

Data are available under the terms of the Creative Commons Attribution 4.0 International license (CC-BY 4.0).

## Author contributions

Conceptualization and methodology, S.; validation, S.; investigation, R.J.H.P.; resources, S.; writing—original draft preparation, S.; writing—review and editing, S., C.F.T. and A.I.P.; visualization, S.; supervision, S. and A.I.P.; funding acquisition, S. and A.I.P.
