# Supplementary material for: Effect of hydrothermal treatment of titanium in high concentration of AgNO 3 solution on surface morphology and roughness
Source: F1000Res. 2022 Feb 23;11:221. [Version 1] doi: 10.12688/f1000research.79542.1 (PMC9672529; doi:10.12688/f1000research.79542.1)
Supplement: Peer review history

# Peer review: Reviewer response for version 1

**DOI:** 10.5256/f1000research.83536.r154122

**Published:** 2022-11-17

**Authors:** John Nicholson

**Affiliations:** 1 Bluefield Centre for Biomaterials, London, UK

The paper reports a useful and well-planned study of the effect of hydrothermal treatment of titanium with silver nitrate, with the aim of developing a new type of implant surface with inherent anti-bacterial properties.

 Surface analysis of the finished metals showed that a non-homogeneous silver coating is deposited on the titanium, which is thicker with higher concentrations of silver nitrate in the treatment solution. Roughness of the surface was almost unchanged by the treatment. The finished coating is a potentially useful modification for clinical application in dental implants.

Is the work clearly and accurately presented and does it cite the current literature?

Yes

If applicable, is the statistical analysis and its interpretation appropriate?

Not applicable

Are all the source data underlying the results available to ensure full reproducibility?

Yes

Is the study design appropriate and is the work technically sound?

Yes

Are the conclusions drawn adequately supported by the results?

Yes

Are sufficient details of methods and analysis provided to allow replication by others?

Yes

Reviewer Expertise:

Dental materials, oral biocompatibility, alloys, cements.

I confirm that I have read this submission and believe that I have an appropriate level of expertise to confirm that it is of an acceptable scientific standard.

# Peer review: Reviewer response for version 1

**DOI:** 10.5256/f1000research.83536.r124776

**Published:** 2022-04-01

**Authors:** Le Thi Bang

**Affiliations:** 1 School of Materials Science and Engineering, Hanoi University of Science and Technology, Hanoi, Vietnam

This research work is the investigation of Ti surface morphology and topography under hydrothermal treatment of Ti in solely AgNO3 at different concentrations for the antibacterial application. Method in this study is facile and effective for surface modification of any implant shape. The results showed that Ag coating composed of mainly metallic Ag and in smaller quantities, Ag oxide with higher agglomeration at higher Ag concentrations. And the coating does not alter the surface roughness significantly.

 The study design is appropriate and the work is technically sound. However, some aspects have to be addressed:

1. There are several grammatical errors needed to be revised, for some examples:

“the absent of a toxic reduction…” should be “the absence of….”

 “This results in silver particulates being deposited on the Ti surface often in nanoscale, thus called silver nanoparticles (AgNPs)”- verb of the sentence is missing.

 “Figures 1 and 2 show the photographs and SEM images” should be “Figures 1 and 2 show the photographs and SEM images, respectively”. In addition, please give some explanation in the text for Fig. 1.

 “The concentrated Ag area was found to be larger in 0.1 mol/L AgNO3 than that in 0.01 mol/L AgNO3. These findings suggest…” should be “The concentrated Ag ,,,, this finding suggests…”

 Further comments:

1. Although the coating layer was analyzed by the EDX mapping, and it seems that the author speculates the phase of the coating. Do the authors confirm the phase identification of the Ti sample and the precipitated powder after hydrothermal treatment by XRD?  
2. Why is the reduction agent needed to be used for the silver nanoparticle coating in previous studies? For example, for a homogeneous coating? What is the positive effect of using solely AgNO _3_ coating to produce the antibacterial property to the Ti implant in comparison to the previous study?
3. In Page 2 the authors stated that “It is known that Ti surface naturally forms thin oxide layer which contain Ti-OH groups on its outmost part” please revise this because the negative charge of Ti-OH was only obtained after treatment in alkali solution (see ^1^)?
4. Did the coating particles just precipitate on the Ti surface or there is any possibility of bonding between the coating and the substrate?
5. In the SEM of the coating sample, the coating particles are not in nanoscale size? Can the author give some possibility of agglomeration of the particles?
6. In page 3, the author stated that “The concentrated Ag area was found to be larger in 0.1 mol/L AgNO3 than that in 0.01 mol/L AgNO3. These findings suggest that at high concentration of AgNO3, the Ag coating tends to be aggregated and thicker, thus the use of a lower concentration might be preferred” please explain why? What is the minimum amount Ag requirement for the antibacterial effect and the maximum Ag amount that is safe for the application? Do the homogenous and entirely cover coating is needed for the application? 

Is the work clearly and accurately presented and does it cite the current literature?

Yes

If applicable, is the statistical analysis and its interpretation appropriate?

Not applicable

Are all the source data underlying the results available to ensure full reproducibility?

Yes

Is the study design appropriate and is the work technically sound?

Yes

Are the conclusions drawn adequately supported by the results?

Yes

Are sufficient details of methods and analysis provided to allow replication by others?

Yes

Reviewer Expertise:

Biomaterials, calcium phosphate based bioceramic, metal implant, surface modification

I confirm that I have read this submission and believe that I have an appropriate level of expertise to confirm that it is of an acceptable scientific standard.

## References

[1] : The effect of heat treatment on bone-bonding ability of alkali-treated titanium. *Biomaterials* .1999;20(5) : 10.1016/S0142-9612(98)90203-4 491-500 10.1016/S0142-9612(98)90203-4 10204992
